# MSM Supplementation Is Associated with Reduced Inflammation and Improved Innate Immune Response following In Vitro LPS-Stimulation in Humans after a Bout of Downhill Running

**DOI:** 10.3390/muscles2020015

**Published:** 2023-05-06

**Authors:** Brian K. McFarlin, Jakob L. Vingren, David W. Hill, Elizabeth A. Bridgeman

**Affiliations:** 1 Applied Physiology Laboratory, University of North Texas, Denton, TX 76203, USA; 2 Department of Biological Sciences, University of North Texas, Denton, TX 76203, USA

**Keywords:** exercise-induced muscle injury, DAMP, Nanostring, exercise recovery

## Abstract

Exercise-induced muscle injury and the subsequent release of Damage-Associated Molecular Patterns (DAMP) result in soreness and inflammation. Dietary supplements may accelerate the rate of recovery by supporting resolution of inflammation. The purpose of this study was to determine if methylsulfonylmethane (MSM) supplementation (30 d prior to exercise and during recovery) altered mRNA expression in LPS-exposed blood leukocytes after a bout of downhill running. Exercise consisted of 60 min of downhill running (−15% grade). Blood (baseline, pre-exercise, 4, 24, 48, and 72 h post-exercise) was diluted (1:10) and combined with LPS (20 µg/mL) for 24 h. Total RNA was isolated from leukocytes and analyzed for 574 immune-associated mRNA (Nanostring nCounter; ROSALIND.BIO). Data were expressed as log2 fold change from baseline for each condition (MSM and placebo). Compared to placebo, MSM supplementation was associated with an improved inflammation response (15 mRNA) and viral immune response (2 mRNA). The largest number of changes were found at 4 and 24 h post-exercise. The key finding in the present study is that MSM supplementation can improve inflammation management and the innate immune response after exercise.

## 1. Introduction

Strenuous exercise can result in peripheral muscle injury and soft tissue damage from a combination of eccentric muscle contractions and oxidative stress [1,2,3,4]. The later inflammatory and immune system response is proceeded by the release of damage-associated molecular patterns (DAMPs). DAMPs initiate a danger response via pattern recognition receptors (PRR) that results in sterile inflammation [5]. Toll-like Receptor 4 (TLR4) is a PRR that is associated with DAMP response [6,7,8,9,10]. DAMPs are remarkably similar in both structure and the response they initiate to pathogen associated molecular patterns (PAMPs). Leukocyte capacity to respond to PAMPs in vitro may provide insight concerning DAMP response in vivo [11,12]. Previous studies from our laboratory and others have successfully used lipopolysaccharide (LPS) exposure to measure secretory response; however, in the present study we took an alternate approach and examined expression of immune-associated mRNA in the LPS-exposed leukocytes following exercise-induced muscle damage. 

Previous researchers have examined a variety of commonly available dietary ingredients with the potential to alter immune response, reduce or resolve sterile inflammation, and improve muscle recovery [13,14,15,16,17,18,19]. Unfortunately, one limitation of previous studies is that often the results are inconclusive because either the supplement is not effective, or the outcome measures are not robust enough to identify an exact effect. In the present study, we have addressed this later limitation using the in vitro LPS exposure model to examine many immune-associated mRNA. Methylsulfonylmethane (MSM) has demonstrated good efficacy as an active sulfur donor contributing to soft tissue healing following injury [18,19]. Specifically, we have reported that MSM in combination with other dietary polyphenols improved muscle recovery following long-distance running [14,16,17]. These conclusions were based on responses of a combination of protein and mRNA biomarkers. The present study extends these findings using a controlled laboratory model of muscle injury, LPS exposure, and mRNA detection. We hypothesized that MSM supplementation would be associated with a change in a subset of mRNA that may be associated with an altered post-exercise recovery profile. The purpose of the present study was to determine if oral MSM supplementation altered whole blood expression of 574 immune-associated mRNA following in vitro LPS exposure during recovery from exercise-induced muscle injury. LPS exposure was used to identify mRNA associated with PAMP/DAMP detection and signaling.

## 2. Results

### 2.1. Exercise-Induced Muscle Damage Response

**Figure 1:**
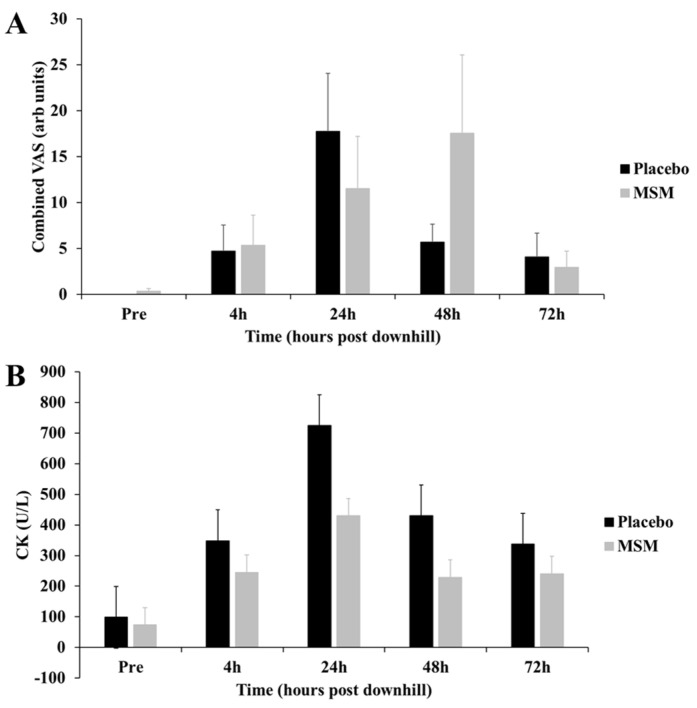
Exercise-Induced Muscle Damage Response. Participants completed six intervals (5 min each) of downhill running on a motorized treadmill (−15% grade, male: 7.0 mph; 11.27 kph and female: 6.0 mph; 9.66 kph). Participants consumed either MSM (3 g/d) or placebo for 30 d prior to EIMD. Subjective soreness and serum creatine kinase were measured at Pre, 4, 24, 48, and 72 h post EIMD. Panel (**A**) total subjective muscle soreness was calculated by measuring bilateral soreness using a visual analog scale at the vastus lateralis and gastrocnemius and summed to yield total subjective soreness. Panel (**B**) skeletal muscle creatine kinase was measured using an enzymatic assay. While neither subjective soreness nor CK response reached significance, there was a trend toward the MSM group producing lower results than placebo.

To characterize the degree of exercise-induced muscle damage (EIMD), we measured serum skeletal muscle creatine kinase response and subjective muscle soreness. We found no significant difference in the quantity of EIMD; however, there was a trend toward the MSM group having a smaller quantity of muscle injury (Figure 1).

### 2.2. Global Summary mRNA Response to EIMD

**Figure 2:**
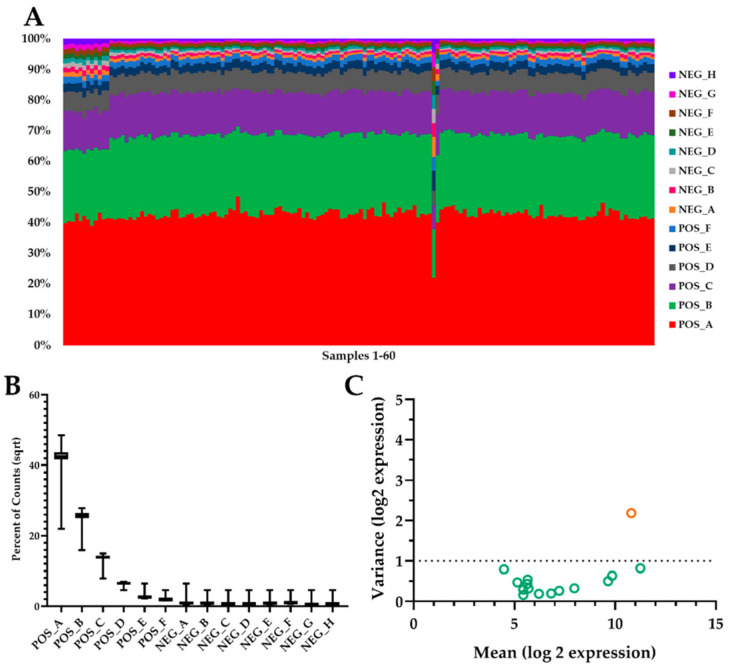
Quality Control Analysis. Panels (**A**,**B**) represent the percentage of eight negative and six positive controls spiked into each sample. Control count expression was within manufacturer guidelines. One sample appeared to be outside of the range of the other samples; however, it was still within manufacturer parameters. Panel (**C**) represents the expression of 15 housekeeper mRNAs chosen for normalization (green circles) and one housekeeping mRNA (orange circle) that was excluded from normalization based on the geNorm algorithm.

One challenge of working with small blood volumes is extracting sufficient total RNA for analysis. Using the Nanostring (Nanostring Human Immunology v2; Seattle, WA) analysis approach, we were able to overcome that common barrier. Our preliminary quality control analysis demonstrated that all controls and housekeeping gene expression were consistent between samples (Figure 2). As well, overall RNA expression was consistent among samples. Of the initial 574 mRNA targets, we found 17 mRNA that were altered by exercise alone or a combination of exercise and supplement (See Appendix A for full names and accession numbers). Using immune response pathways and previously published research, we were able to annotate the significant mRNA to either inflammation (15 mRNA) or viral immune response (2 mRNA). Another interesting observation was that MSM supplementation resulted in significant differences from placebo at 4 and 24 h post-exercise. No significant MSM effects were observed at either 48 or 72 h post-exercise.

### 2.3. Inflammation-Associated mRNA

**Figure 3:**
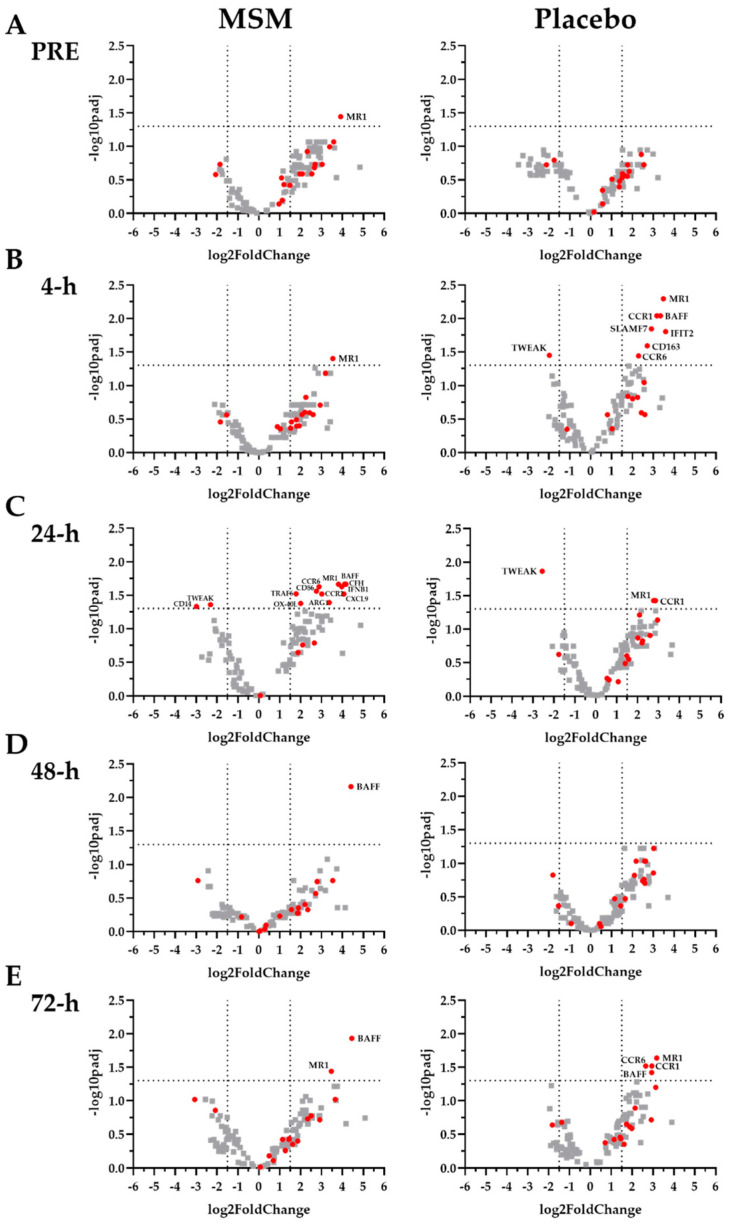
Volcano plots of differentially expressed genes in each supplement condition at each time point. Values represent log2 fold change and significance shown as—log10padj. Padj was set at <0.05. Panels (**A**) PRE, (**B**) 4 h, (**C**) 24 h, (**D**) 48 h. and (**E**) 72 h, represent differential gene expression of MSM and placebo relative to baseline. Values represent time points following exercise-induced muscle injury from downhill running.

**Figure 4:**
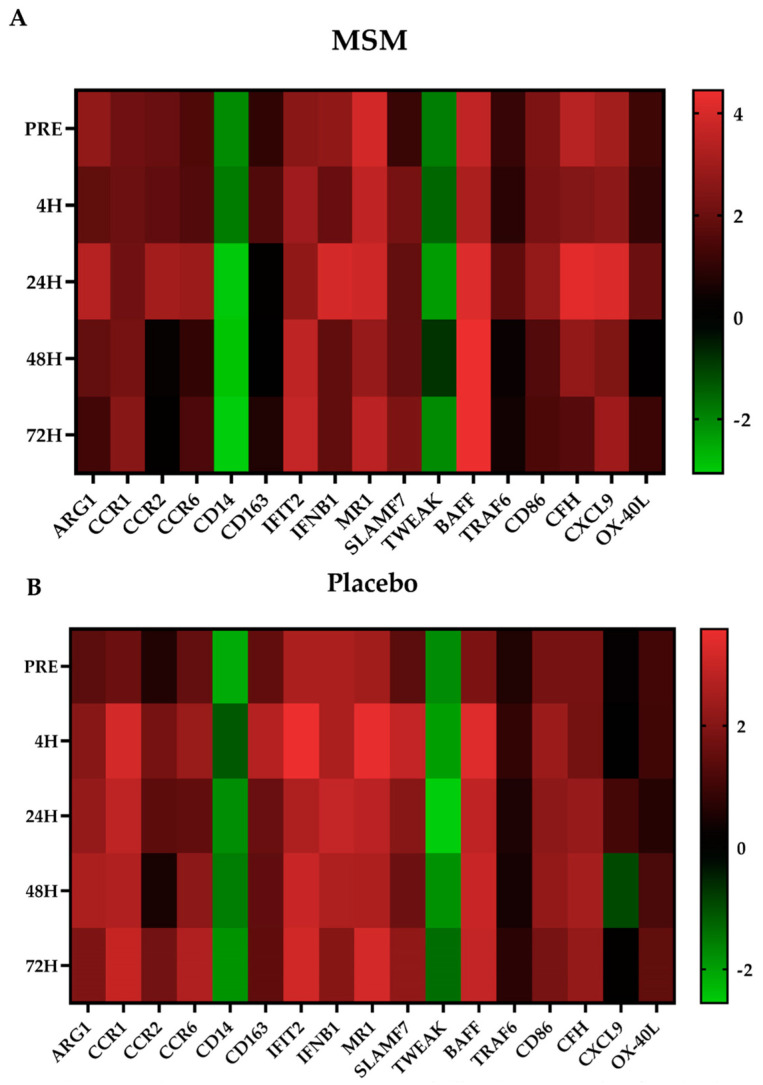
Panels (**A**,**B**) represent heat maps of differential expression for MSM (**A**) and placebo (**B**) at all time points expressed as log2 fold change. Values represent time points following exercise-induced muscle injury from downhill running.

We found 15 mRNA (CD14, CD163, CXCL9, CFH, CD86, CCR1, CCR2, CCR6, IFIT2, SLAMF7, MR1, ARG1, IFBN1, TRAF6, and TWEAK), which have been previously linked to various aspects of the inflammatory response, whose expression was significantly altered with MSM compared to placebo. All the significant MSM effects were found at 4 h (Figure 3B and Figure 4; CCR1, CCR6, CD163, IFIT2, and SLAMF7) and/or 24 h (Figure 3C and Figure 4; CCR2, CCR6, CD14, IFNB1, MR1, and TRAF6) post-exercise. In each case, MSM supplementation either increased or decreased the observed effect in the placebo condition.

### 2.4. Viral Immune Response mRNA

We found two mRNA (BAFF and OX-40L), which are associated with viral immune response, whose expression was altered with MSM compared to placebo. In the present study, MSM was associated with an increased expression of BAFF and OX-40L at 24 h post-exercise (Figure 3C and Figure 4).

## 3. Discussion

The key finding of the present study was that the MSM supplementation altered expression of several mRNA that are associated with inflammation and with the viral immune response following exercise-induced muscle injury. Using in vitro LPS exposure allowed us to uniquely identify mRNA whose change was linked to DAMP detection and signaling in the context of skeletal muscle injury. It is interesting that despite recovery from exercise being a multi-day process, MSM-associated changes were observed only early in recovery (i.e., 4 and 24 h post-exercise). This discussion focuses on those time points because MSM does not appear to affect late recovery (i.e., 48 and 72 h post-exercise). It is common that muscle injury from exercise is a biphasic response with the second peak in that response being caused by secondary damage caused by the immune response to DAMP. Given the observed changes, it is reasonable to speculate that MSM supplementation may have reduced the severity of secondary damage despite a similar quantity of primary damage between MSM and placebo conditions. The implications of the MSM supplementation on post-exercise inflammation and viral immune response are discussed in subsequent sections. 

CD14, CD163, CXCL9, CFH, CD86, CCR1, CCR2, CCR6, and IFIT2 are all associated with immune response to DAMP/PAMP and later inflammation [20,21,22,23,24,25,26,27,28,29]. Elevated CD14 expression has been consistently reported for up to 24 h after a strenuous bout of exercise [20,21] and in patients with chronic sepsis [30]. CD14 is a key cell-surface receptor that mediates monocyte response to LPS [10,31]. In the present study, we found reduced CD14 expression in both conditions; however, the reduction was greater for MSM at 24, 48, and 72 h post-exercise. Given the role of CD14 in sterile inflammation, it is reasonable to speculate that the observed changes are associated with reduced inflammation and an increased speed of recovery when MSM is consumed. CD163 is positively correlated to exercise intensity or the degree of muscle injury [22,23]. Earlier studies reported changes in CD163 at 4 h post-exercise, and sustained exposure to LPS results in an increase in soluble CD163 via monocyte shedding [32,33]. In the present study, we observed an increase in CD163 expression for both conditions at 4 h; however, the change was smaller with MSM. Lower CD163 expression with MSM may have resulted in less CD163 shedding compared to placebo. Similar to CD163 expression, at 4 h we observed that, compared to placebo, MSM supplementation was associated with a blunting of the increase in CCR1, CCR6, and IFIT2 expression. A small increase in expression of CCR1, CCR2, and CCR6 persisted at 24 h. Given their respective roles in the mediation of PAMP response, a sustained elevation with MSM may reflect a beneficial response that allows for a more efficient immune response to additional DAMP signals. CXCL9 is a chemokine that is associated with skeletal muscle damage [34] and confers anti-microbial activity in the presence of IFN-gamma [35]. CFH (Factor H) down-regulates neutrophils and monocytes via a complement pathway [36] and may have anti-inflammatory activity [37]. CD86 is a costimulatory molecule that facilitates antigen presentation and immune response to various PAMP and has been linked to exercise response [38]. We observed an increase in CXCL9, CFH, and CD86 mRNA expression in MSM but not placebo at 24 h, which may be a beneficial adaptation. Thus, the MSM-associated changes in CD14, CD163, CXCL9, CFH, and CD86 expression may reflect a potential for improved recovery from exercise-induced muscle injury given their role with DAMPs and inflammation.

In the present study, MSM resulted in a reduced expression of SLAMF7 at 4 h and an increased expression of MR1 at 24 h compared to placebo. SLAMF7 increases tissue macrophage response to inflammation and is elevated in chronic autoimmune disease [39], while MR1 protects cardiac muscle from inflammatory injury [40]. Given the biological actions of SLAMF7 and MR1, MSM supplementation may be associated with reduced post-exercise inflammation and resistance to secondary inflammatory injury. 

ARG1 has been shown to increase while IFBN1 and TWEAK have been shown to decrease following long-distance running [23,41]; all three are associated with inflammatory response. Regardless of timing, ARG1 and TWEAK exert anti-inflammatory effects [23,42], while IFBN1 is a key part in LPS signaling [43]. In the present study, we observed that MSM supplementation was associated with a greater increase in ARG1 and IFBN1 expression than placebo at 24 h. Further, MSM was associated with a smaller decrease in TWEAK expression compared to placebo at both 4 h and 24 h. Our findings for ARG1, IFBN1, and TWEAK support the notion that MSM supplementation reduces the capacity for inflammation compared to placebo, which may translate to a more rapid muscle recovery. 

Our laboratory has previously demonstrated that a combination of MSM, curcumin, and pomegranate supplements was associated with reductions in TRAF6 following intense running of different distances [14,17]. In the present study, where only MSM was used, supplementation was associated with a greater TRAF6 expression than placebo at 24 h. It is reasonable to speculate that the single MSM supplement used in the present study exerted a different action than the combination supplement we have previously tested.

While most of the mRNA whose expression was altered with MSM following exercise were associated with inflammation, we also found two mRNA (BAFF and OX-40L) whose expression has been previously linked to viral immune response [44,45,46]. The immune response following exercise-induced muscle damage is more effective if there is no post-exercise infection. Our laboratory and others have previously demonstrated that post-exercise upper respiratory tract infection is particularly challenging to exercise recovery [47,48]. In the present study, MSM increased BAFF and OX-40L expression at 24 h compared to placebo. Based on the effects of MSM supplementation on BAFF and OX-40L, and the purported effects of MSM supplementation on these two mRNA, it appears that MSM supplementation may reduce the risk of viral infection and upper respiratory tract infection after exercise. More research is needed to evaluate the implications of the MSM-induced alterations in BAFF and OX-40L.

One limitation in the present study was the small sample size (N = 12). We overcame this limitation by using the Nanostring analysis approach, which allows for the generation of large amounts of data despite small sample sizes [14,49]. A second limitation was that the present study focused only on mRNA biomarkers. First, it would not have been feasible to include matching protein targets for the 574 mRNA that we measured. But the present study did identify a candidate set of 17 mRNA for which the protein targets could be evaluated further in future studies. Men and women may differ in their response to muscle injury [50,51]; however, regardless of the potential difference, they both have a DOMS response. If anything, women may have a less pronounced DOMS response than men, which would be consistent with a lower statistical power. It was never a goal of the present study to identify sex-specific effects, but rather overall effects that could guide future studies. While we acknowledge that some individuals may disagree with this approach, we do want to direct attention to the fact that despite sex differences in the magnitude of the DOMS response, we still found 17 mRNA that reached significance with a moderate effect size. In the future, we plan to conduct a larger study to confirm the mRNA identified in this study, identify additional mRNA, and hopefully better characterize the effect of MSM treatment in different sexes. It is certainly our hope that other researchers will use our findings as a guide and direction for future work in this area. The purpose of this study was to focus on systemic changes; however, it is plausible that had we examined skeletal muscle, we might have observed a different response. Despite these limitations, we believe that the findings of the present study will inform future studies involving post-exercise biomarker targets.

We identified 17 mRNA that were significantly changed to a greater degree with MSM than with placebo supplementation. The analysis of LPS-stimulated mRNA expression adds another dimension to the overall analysis given the similarities between DAMPs and PAMPs. The observed changes can be linked back to pathways associated with inflammation and viral immune response. Both pathways have implications in muscle recovery after exercise-induced injury. In the present study, we noted that MSM supplementation resulted in the most significant changes in mRNA associated with inflammation at 4 h and in mRNA associated with viral immunity at 24 h. Further interpretation of the evaluated time points indicates that MSM was associated with improved inflammation and viral immune response during early recovery (≤24 h after injury-inducing exercise). 

## 4. Materials and Methods

### 4.1. Participants

**Figure 5:**
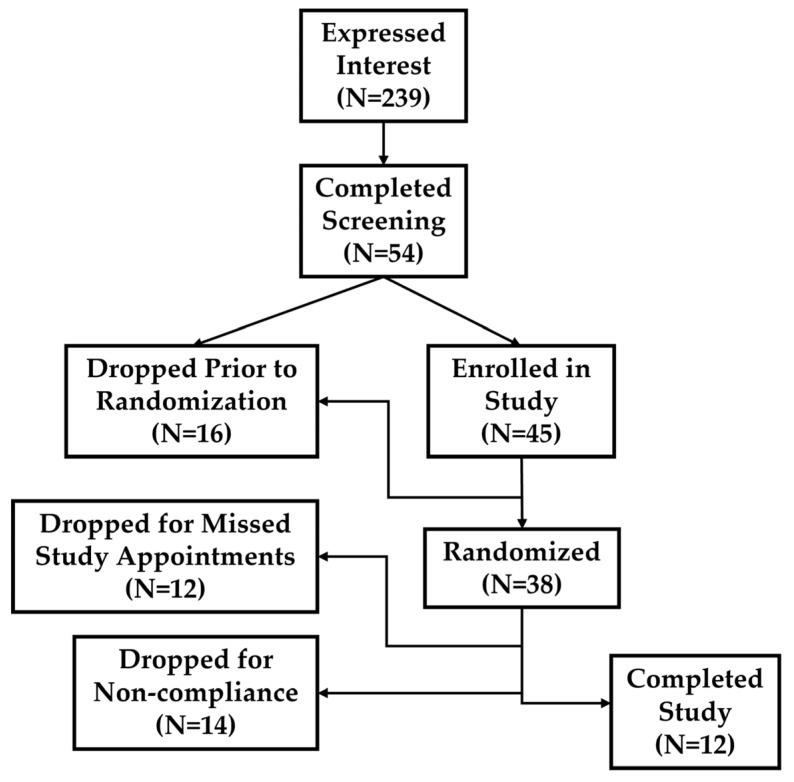
Consort diagram and flow of participants through the present study. There were 239 individuals initially interested in the study. Based on experimental inclusion/exclusion criteria and participant attrition, a total of 12 participants completed the study protocol.

All subjects gave their informed consent for inclusion before they participated in the study. The study was conducted in accordance with the Declaration of Helsinki, and the protocol was approved by the University of North Texas Institutional Review Board for the Protection of Human Subjects in Research (Project Identification Code #14375). Interested participants gave written informed consent to participate in the study. Young (18–35 y), physically active men and women were recruited to participate in the present study. As this study was not focused on body composition as an outcome measure, body composition was not tested and thus may be a potential limitation. A study consort diagram is presented in Figure 5. Complete participant characteristics are presented below in Table 1. 

### 4.2. Supplement Conditions

The 12 participants were randomly distributed to one of two supplement conditions using double-blind procedures: methylsulfonylmethane (MSM 3 g/d; Bergstrom Nutrition; Vancouver, WA, USA) or placebo (rice flour). Capsules were designed such that each participant took three capsules per day from a blister pack format to aid in supplement compliance. Supplements were consumed daily for 30 d prior to the downhill running trial. Participants were asked to promptly report missed doses to the study staff and compliance with the study protocol was found to be 92%.

### 4.3. Statistical Sample Size Analysis

The present study was designed to pilot the effect of MSM on LPS-stimulated mRNA expression following a bout of downhill running. Generally, pilot studies are small in nature and targeted enrollment is based on the effect size of a given outcome measure. The present study was originally developed to measure a different set of research questions than the present study. Several years after this study was originally completed, we added Nanostring analysis capability to our laboratory and learned that it is useful for small sample size studies [14,16,17]. At that time, we re-evaluated archived samples from previous studies and identified the present samples as a suitable target for Nanostring analysis. We fully acknowledge that men and women differ in their physiological response to exercise and while we cannot draw conclusions on sex-specific responses, we speculate that the present findings may direct future research designed to compare sex differences. Despite the inability to separate effects relative to sex, we still identified 17 mRNA that reached significance and had high statistical power (>0.80). The remaining mRNA that did not reach significance had low statistical power. While a larger sample size may reveal additional mRNA affected by MSM, it does not negate the observed findings.

### 4.4. Blood Sample Collection

Venous blood samples were collected before and after performance of a standardized running trial: baseline (prior to supplementation) and at 30 d (see below for details of downhill running trial). Samples were collected into three evacuated tubes, one containing EDTA, a second containing lithium heparin, and the third containing a clot activator. The EDTA tube was used to measure the complete blood count (CBC) and 3-part differential using an automated analyzer (CDS Medonic; Plantation, FL, USA). This test confirmed that participants were not anemic, sick, may not be vitamin/mineral deficient, or dehydrated at the time of testing. While this study was focused on systemic responses of mixed leukocyte populations, future studies may seek to localize the observed effects to specific leukocyte subsets. The heparin tube was used to complete LPS-stimulation mRNA production. Serum was collected from the clot activator tube and frozen at −80 °C until analysis for skeletal type creatine kinase (CK) concentration using an enzymatic assay (Pointe Scientific; Canton, MI, USA) and an automated chemistry analyzer (Chemwell 2910; Palm City, FL, USA). CK was used as a biological index of muscle damage.

### 4.5. Downhill Running Trial

Between 6:00 am and 10:00 am, participants reported to the laboratory to complete the experimental exercise trial that consisted of six intervals (5 min each) of downhill running (−15% grade) on a motorized treadmill. Participants were allowed a 2 min passive recovery between each exercise bout. The initial treadmill speed (male: 7.0 mph; 11.27 kph and female: 6.0 mph; 9.66 kph) was adjusted individually to ensure that every participant demonstrated an exaggerated eccentric running motion. The downhill running motion was demonstrated to the participants prior to their first interval and then study staff coached them on their form throughout the session. Venous blood samples were collected prior to exercise (PRE), and 4, 24, 48, and 72 h post-exercise. 

### 4.6. Subjective Muscle Soreness

Bilateral subjective soreness was measured at the vastus lateralis (lateral upper leg, midpoint between knee and hip) and gastrocnemius (midpoint of muscle belly between ankle and knee). At PRE, a mark was placed on the skin using a permanent marker. A 10 N force was applied to the marked location and participants were asked to rate their soreness on an unlabeled 10 cm visual analog scale (VAS) as described previously [15]. The four VAS scores were summed to yield total subjective soreness. 

### 4.7. LPS-Stimulation

In Vitro exposure of blood leukocytes to LPS mimics how these cells would respond to DAMP in vivo [38,52]. Heparinized whole blood was diluted (1:10) with phosphate buffered saline (PBS) and added to individual wells (950 mL) of a 24-well tissue culture plate. Diluted LPS (50 mL) was added to each well from stock to yield a final LPS dose of 20 µg/mL. This LPS dose was determined from previous research in our laboratory and from pilot testing for the present study (data not shown). Blood/LPS cultures were incubated for 24 h in a 37 °C, 5% CO_2_, humidified incubator. After stimulation, supernatant was removed and plates were sealed with parafilm and stored in an evacuated, desiccated plastic freezer bag at −80 °C until mRNA extraction and analysis. 

### 4.8. RNA Isolation from Cell Pellets 

Plates were removed from the freezer and thawed at room temperature for 1 h prior to RNA extraction using a commercially available kit (Qiagen RNeasy Mini Kit for Animal Cells; Hilden, Germany) on an automated sample processing instrument (QIACube; Qiagen; Hilden, Germany). Briefly, thawed cell pellets were lysed with RLT1 buffer (350 mL) for 1 h on a shaking incubator set to 60 °C. The resultant solution was transferred to the Qiagen-specific processing tubes and loaded into the QIACube. Isolated RNA was further processed using a concentrator (Zymo Clean & concentrator kit; Irvine, CA, USA) to adjust to a concentration of 10 ng/mL that is needed for the Nanostring assay. Total RNA concentration was measured using a fluorometric method (Invitrogen Qubit 2.0; Waltham, MA, USA).

### 4.9. Nanostring Analysis

Total RNA samples were processed using a commercially available, 574-plex Immunology panel (Nanostring Human Immunology v2; Seattle, WA, USA). Following an 18 h hybridization at 65 °C in a thermocycler, samples were loaded into individual lanes of a Nanostring nCounter Sprint cartridge and inserted into the Nanostring Sprint instrument. 

### 4.10. Statistical Analysis

Raw RCC files (standard file type generated by the Nanostring instrument) were obtained from the Sprint instrument and analyzed by ROSALIND^®^ (https://rosalind.onramp.bio/ (accessed on 12 April 2021); San Diego, CA, USA) with a HyperScale architecture developed by ROSALIND, Inc. Read distribution percentages, violin plots, identity heatmaps, and sample multidimensional plots were generated as part of the quality control step. Normalization values were calculated using criteria provided by Nanostring. ROSALIND^®^ follows the nCounter^®^ Advanced Analysis protocol of dividing counts within a lane by the geometric mean of the normalizer probes from the same lane. Housekeeping probes that were used for normalization were selected based on the geNorm algorithm as implemented in the NormqPCR R library [53]. Fold changes and significance score (*p*-value) were calculated using the fast method as described in the nCounter^®^ Advanced Analysis 2.0 User Manual (Nanostring). No effects of sex were noted and thus groups were collapsed into single participant populations.

Log2 fold change for each time point compared to baseline was calculated separately for MSM and placebo; changes were normalized to a 0 center to determine direction of exercise effects (i.e., up- vs. downregulated). Differential expression analysis was completed at the resulting 5 time points (PRE, 4, 24, 48, and 72 h post-exercise). Gene clustering (based on direction and type of all signals on a pathway, the position, role, and type of every gene) of differentially expressed genes was determined using the Partitioning Around Medoids method and the fpc R library (Hennig C. Cran-package fpc. https://cran.r-project.org/web/packages/fpc/index.html. 2019) (accessed on 12 April 2021). Significant clusters were identified and then used in a ROSALIND^®^ Meta-Analysis to identify specific mRNA that were different in MSM compared to placebo. Significant *p*-values (*p* < 0.05) were adjusted for multiple genetic comparisons using the Benjamini–Hochberg method of estimating false discovery rates [54]. 

### 4.11. Data Interpretation and Presentation

Log2 fold change for mRNA is presented in a series of standard volcano plots. Care was taken to identify mRNA that were significantly changed. The immunologic pathway with which a particular mRNA was associated was determined using standard Nanostring pathway annotations and previously published studies indexed in PubMed. An individual literature search was completed on each of the 17 mRNA that were affected by the exercise to link the observed response to previously published results. The results of the present study confirm previous reports that injury-provoking exercise affects the expression of a number of mRNA (CD14, CD163, CD86, IFIT2, MR1, ARG1, IFBN1, and TWEAK), and we have also demonstrated that the exercise affects CXCL9, CFH, CCR1, CCD2, CCR6, SLAMF7, TRAF6, BAFF, and OX-40L, and that supplementation with 3 g/d MSM has beneficial effects on each of these.

## Figures and Tables

**Table 1:** Participant characteristics.

Variable	Placebo (N = 7)	Supplement (N = 5)
Sex (F)	2	4
(M)	5	1
Age (Y)	22 ± 1	20 ± 1
Height (cm)	177.6 ± 3.8	166.9 ± 3.8
Weight (kg)	72.9 ± 4.9	64.8 ± 5.1
BMI (kg/m^2^)	33.1 ± 2.2	29.4 ± 2.3

Values represent the mean ± standard error of the mean (SEM). Variables were compared between methylsulfonylmethane (MSM 3 g/d; Bergstrom Nutrition; Vancouver, WA) and Placebo (rice four) in healthy adults 30-d prior to a downhill running trial. No significant differences (*p* > 0.05) detected for participant characteristics between condition groups.

## Data Availability

The data presented in this study are available on request from the corresponding author.

## Appendix A

**Table A1:** Gene Abbreviations.

Abbreviation	Full Name	Accession ID
ARG1	Arginase	NM_000045.2
BAFF	Tumor necrosis factor (ligand) superfamily, member 13b	NM_006573.4
CCR1	Chemokine (C-C motif) receptor 1	NM_001295.2
CCR2	Chemokine (C-C motif) receptor 2	NM_001123041.2
CCR6	Chemokine (C-C motif) receptor 6	NM_031409.2
CD14	CD14 molecule	NM_000591.2
CD86	CD86 molecule	NM_175862.3
CD163	CD163 molecule	NM_004244.4
CFH	Complement factor H	NM_001014975.2
CXCL9	Chemokine (C-X-C motif) ligand 9	NM_002416.1
IFIT2	Interferon-induced protein with tetratricopeptide repeats 2	NM_001547.4
IFNB1	Interferon, beta 1, fibroblast	NM_002176.2
MR1	Major histocompatibility complex, class I-related	NM_001531.2
OX-40L	Tumor necrosis factor (ligand) superfamily, member 4	NM_003326.2
SLAMF7	SLAM family member 7	NM_021181.3
TRAF6	Tumor necrosis factor receptor-associated factor 6	NM_145803.1
TWEAK	Tumor necrosis factor (ligand) superfamily, member 12	NM_003809.2
